# Interaction of the Left–Right Somatosensory Pathways in Patients With Thalamic Hemorrhage: A Case Report

**DOI:** 10.3389/fnhum.2021.761186

**Published:** 2021-11-01

**Authors:** Daisuke Ishii, Kiyoshige Ishibashi, Kotaro Takeda, Hiroshi Yuine, Satoshi Yamamoto, Yuki Kaku, Arito Yozu, Yutaka Kohno

**Affiliations:** ^1^Center for Medical Sciences, Ibaraki Prefectural University of Health Sciences, Inashiki-gun, Japan; ^2^Department of Cognitive Behavioral Physiology, Chiba University Graduate School of Medicine, Chiba, Japan; ^3^Department of Physical Therapy, Ibaraki Prefectural University of Health Sciences Hospital, Inashiki-gun, Japan; ^4^Faculty of Rehabilitation, School of Healthcare, Fujita Health University, Toyoake, Japan; ^5^Department of Occupational Therap, School of Health Sciences, Ibaraki Prefectural University of Health Sciences, Inashiki-gun, Japan; ^6^Department of Physical Therapy, School of Health Sciences, Ibaraki Prefectural University of Health Sciences, Inashiki-gun, Japan

**Keywords:** stroke, somatosensory evoked potentials, neural plasticity, paired somatosensory evoked potentials, thalamic hemorrhage

## Abstract

Neural plasticity compensates for the loss of motor function after stroke. However, whether neural plasticity occurs in the somatosensory pathways after stroke is unknown. We investigated the left–right somatosensory interaction in two hemorrhagic patients using a paired somatosensory evoked potentials (p-SEPs) recorded at CP3 and CP4, which was defined as an amplitude difference between the SEPs of paired median nerve stimulations to both sides and that of single stimulation to the affected side. Patient 1 (61-year-old, left thalamic hemorrhage) has a moderate motor impairment, severe sensory deficit, and complained of pain in the affected right upper limb. Patient 2 (72-year-old, right thalamic hemorrhage) had slight motor and sensory impairments with no complaints of pain. Single SEPs (s-SEPs) were obtained by stimulation of the right and left median nerves, respectively. For paired stimulations, 1 ms after the first stimulation to the non-affected side, followed by a second stimulation to the affected side. In patient 1, a s-SEP with stimulation to the non-affected side and a p-SEP were observed in CP4. However, a s-SEP was not observed in either hemisphere with stimulation to the affected side. On the other hand, in patient 2, a s-SEP in CP3 with stimulation to the non-affected side and in CP4 with stimulation to the affected side were observed; however, a p-SEP was not observed. In addition, to investigate the mechanism by which ipsilateral median nerve stimulation enhances contralateral p-SEP in patient 1, we compared the SEP averaged over the first 250 epochs with the SEP averaged over the second 250 epochs (total number of epochs recorded: 500). The results showed that in the patient 1, when the bilateral median nerve was stimulated continuously, the habituation did not occur and the response was larger than that of the s-SEP with unilateral median nerve stimulation. In the current case report, the damage to the thalamus may cause neuroplasticity in terms of the left–right interaction (e.g., left and right S1). The somatosensory input from the affected side may interfere with the habituation of the contralateral somatosensory system and conversely increase the response.

## Introduction

The human nervous system has acquired some adaptive responses, defined as “hyper-adaptability,” that are activated with significant changes in the internal environment (it does not work under normal conditions). For example, neural plasticity in the central nervous system plays an important role in the recovery of motor paralysis caused by corticospinal tract damage. Previous studies on spinal cord-lesioned monkeys showed that the sprouting of midline-crossing axons in the corticospinal tract occurs in the spinal cord rostral to the lesion, and the sprouting was associated with improvement in hand function and locomotion (Courtine et al., 2005; Rosenzweig et al., 2010). In addition, the functional motor representation maps around the damage and remote cortical regions change with rehabilitative motor training after focal damage in the forelimb movement area of the motor cortex (Nudo et al., 1996; Frost et al., 2003; Ramanathan et al., 2006; Barbay et al., 2013). These studies suggest that an unusual neural route is created, which then controls the paretic limb (Murata et al., 2015; Isa, 2017; Yamamoto et al., 2019; Kato et al., 2020). Conversely, no compensatory mechanism for sensory information processing after damage to the somatosensory pathway has been identified.

Neurons in the unilateral S1 receive sensory information from the contralateral brain through interhemispheric transfer (Iwamura et al., 1994, 2001, 2002; Nihashi et al., 2005). Recently, three previous studies, including our study, investigated interactions between contralateral and ipsilateral activations using a paired median nerve somatosensory evoked potential (p-SEP) protocol in a healthy human (Ragert et al., 2011; Brodie et al., 2014; Ishii et al., 2021). In this protocol, peripheral stimulation of the unilateral median nerve preceded the stimulation of the other median nerve with some interstimulus intervals (ISIs). When left–right interaction is presented, the SEP evoked by the stimulation to the unilateral median nerve is modulated by interference of the stimulation to the median nerve on the other side. Ragert et al. (2011) and Brodie et al. (2014) showed that interhemispheric inhibitory interactions in the S1 occur between two hemispheres *via* the corpus callosum in a critical time interval of 20–25 or 15–35 ms after median nerve stimulation. However, we recently investigated the effect of ISIs (1–100 ms) on p-SEPs in more detail and concluded that no interaction occurs between left and right somatosensory pathways in healthy subjects with any ISI (Ishii et al., 2021). We further demonstrated that no interaction occurs between the bilateral somatosensory pathways of healthy subjects with short ISI conditions (<5 ms), which indicates that there is also no left–right transmission of a small number of synapses in the pathways (Ishii et al., 2021). In stroke patients, in whom large-scale changes in neural networks occur, the existence of a left–right interaction of somatosensory pathways at a level other than the corpus callosum has not been established. However, left–right somatosensory interaction at a level other than the corpus callosum might contribute to recovery from hypoesthesia. This is because when the somatosensory pathways are damaged at the subcortical level, such as in the thalamus, left–right interaction through the corpus callosum may not contribute to the recovery of hypoesthesia.

We hypothesized that the neural connectivity formed by a small number of synapses in the left–right interaction of the somatosensory pathway would be present in patients with recovered sensory impairment. Therefore, we investigated the interaction of the left–right of somatosensory pathways in two patients with thalamic hemorrhage using the p-SEPs protocol under short ISI (Ragert et al., 2011; Brodie et al., 2014; Ishii et al., 2021).

## Case Description

This study was reviewed and approved by the Ibaraki Prefectural University of Health Sciences Review Board (approval nos. 893 and e278). The patients provided their written informed consent to participate in this study.

### Timeline for Clinical and Laboratory Findings

Table 1 shows the clinical episodes and evaluation results from the onset of symptoms to the time of SEP recording.

**TABLE 1:** Clinical and laboratory findings on patients 1 and 2.

Patient 1		Patient 2	
Days	Clinical and laboratory findings	Days	Clinical and laboratory findings
0 (onset)	Admission to the acute care hospital due to right upper and lower limb paresis, facial paresis, and speech disturbance.	0 (onset)	Admission to the acute care hospital due to left upper and lower limb paresis.
2	Starting the acute rehabilitation (occupational, physical, and speech therapy). Brunnstrom stage: 2 (arm), 2 (hand), and 2 (leg) Deep sensations: severe (thumb-localizing test: 3) Superficial sensation: severe		Starting the acute rehabilitation (occupational, physical, and speech therapy). Brunnstrom stage: 2 (arm), 3 (hand), and 2 (leg) Deep sensations: severe Superficial sensation: severe
33	Transferred to a convalescent rehabilitation hospital. Brunnstrom stage: 3 (arm), 3–4 (hand), and 3–4 (foot) Deep sensations: severe (thumb-localizing test: 2) Superficial sensation: severe Pain: affected upper limb	20	Transferred to a convalescent rehabilitation hospital. Brunnstrom stage: 3 (arm), 4 (hand), and 5 (foot) Deep sensations: mild Superficial sensation: mild
107 (SEP recording)	Brunnstrom stage: 3 (arm), 4 (hand), and 4 (foot) Deep sensations: severe Superficial sensation: severe Pain: affected upper limb	152 (SEP recording)	Brunnstrom stage: 5 (arm), 5 (hand), and 4 (foot) Deep sensations: mild Superficial sensation: mild

### Patient 1

**FIGURE 1:**
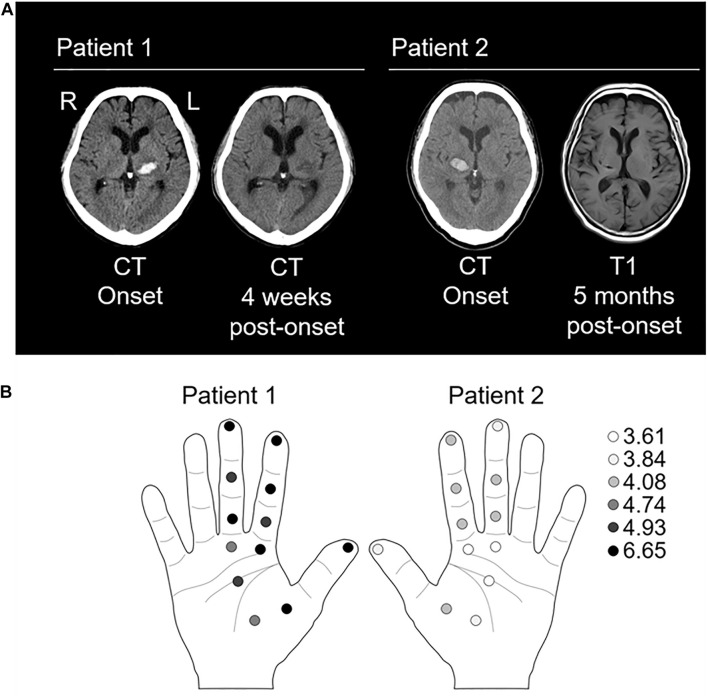
Evaluation of lesioned site and tactile threshold. **(A)** *Left panel*: the computed tomography (CT) images of patient 1 at onset and 4 weeks post-onset show a high-and low-density area in the left thalamus, respectively. *Right panel*: the CT image at onset and T1-weighted magnetic resonance image at 5 weeks post-onset in patient 2 show a high-density area and a low signal-intensity area in the right thalamus, respectively. **(B)** Left panel showing tactile thresholds in patient 1. Right panel showing tactile thresholds in patient 2. The monofilaments of 20 different sizes (1.65–2.83, 3.22–3.61, 3.84–4.31, 4.54–6.45, and 6.65 expressed as the log of the bending force in mg) were applied to the index finger (three points), mother finger (one point), middle finger (three points), and palm (five points). Normal: 1.65–2.83; diminished light touch: 3.22–3.61; diminished protective sensation: 3.84–4.31; loss of protective sensation: 4.56–6.45; deep pressure sensation: 6.65; and unmeasurable: >6.65 (Bell-Krotoski and Tomancik, 1987; Jerosch-Herold, 2005).

A 61-year-old right-handed Japanese man was admitted to the acute care hospital due to right upper and lower limb paresis, facial paresis, and speech disturbance. On admission, the Glasgow Coma Scale (GCS) for the eye-opening, verbal, and motor responses was 3, 5, and 6 points, respectively. Head computed tomography (CT) showed a high-density area in the left thalamus (Figure 1A). On the second day post-onset, acute rehabilitation (occupational, physical, and speech therapy) was initiated. The physical examination revealed the following findings: right upper and lower limb paresis (Brunnstrom stage 2 for arm, 2 for hand, and 2 for leg), and severe hypoesthesia with deep (thumb-localizing test: 3) and superficial sensation. The patient was subsequently transferred to a convalescent rehabilitation hospital at 4 weeks post-onset. Evaluation at the time of transfer showed a GCS of 15 points, paralysis of the right upper and lower limbs (Brunnstrom stage 3 for arm, 3–4 for hand, and 3–4 for foot), severe hypoesthesia with deep sensation (thumb-localizing test: 2), and superficial sensation. In addition, he complained of pain in the right upper limb (affected side).

### Patient 2

A 72-year-old right-handed Japanese man was admitted to the acute care hospital due to left upper and lower limb paresis. On admission, the GCS for the eye-opening, verbal, and motor responses was 4, 5, and 6 points, respectively. Head CT showed a high-density area in the right thalamus (Figure 1A). On the day of onset, acute rehabilitation (occupational, physical, and speech therapy) was initiated. The physical examination revealed the following findings: left upper and lower limb paresis (Brunnstrom stage 2 for arm, 3 for hand, and 2 for leg), and severe hypoesthesia with deep and superficial sensations. The patient was subsequently transferred to a convalescent rehabilitation hospital at 20 days post-onset. Evaluation at the time of transfer showed paralysis of the left upper and lower limbs (Brunnstrom stage 3 for arm, 4 for hand, and 5 for foot), slight hypoesthesia with deep and superficial sensations, and no complaints of pain in the left upper limb (affected side).

### Physical Examination at SEP Recording

On the 107th (patient 1) or 152th (patient 2) day of onset, we performed a physical examination using the Brunnstrom stage (for paresis), sense of passive movement and position sense of thumb (for deep sensations), and Semmes–Weinstein Monofilament (SWM) test (Sakai Medical, Tokyo) for superficial sensation as previously described (Mima et al., 2004). The SWM applies stimulation with constant pressure and evaluates tactile thresholds using several levels of stimulus intensity (Bell-Krotoski et al., 1995). Briefly, monofilaments of 20 different sizes (1.65–2.83, 3.22–3.61, 3.84–4.31, 4.54–6.45, and 6.65 expressed as the log of the bending force in mg) were applied to the index finger (three points), mother finger (one point), middle finger (three points), and palm (five points) (Figure 1B).

### Somatosensory Evoked Potential Recording

**FIGURE 2:**
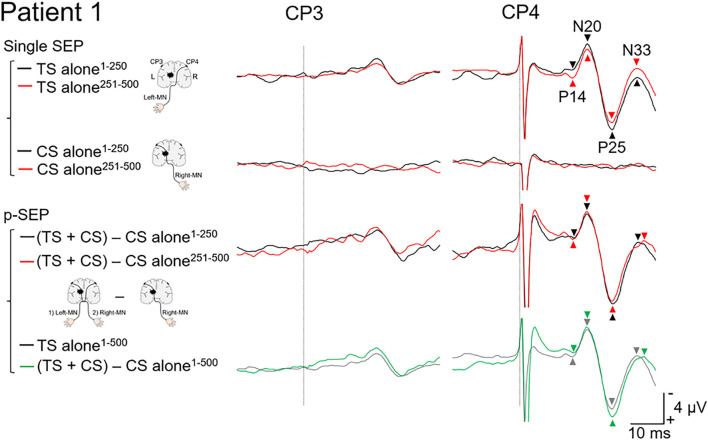
Paired median nerve somatosensory evoked potential in patient 1. *Left panels*: to investigate the effect of the conditioning stimulus (CS) on the SEP induced by the test stimulus (TS), the single median nerve SEPs (single SEP) (right-MN: CS alone), and the paired median nerve SEPs (p-SEP) were recorded at the CP3 and CP4 electrodes (Ragert et al., 2011). In the p-SEP recording, the right-MN stimulus (CS) was performed 1 ms after the left-MN stimulus (TS). *Right panels*: all SEP waveforms at CP3 and CP4 from patient 1. We compared the SEP averaged over the first 250 epochs with the SEP averaged over the second 250 epochs (total number of epochs recorded: 500). Black line: the SEP averaged over the first 250 epochs. Red line: the SEP averaged over the second 250 epochs. Gray line: the SEP averaged over all epochs (500 epochs) (TS alone condition). Green line: the SEP averaged over all epochs (500 epochs) [(TS + CS) − CS alone condition]. Dotted line: the time duration of stimulation. MN, median nerve.

**FIGURE 3:**
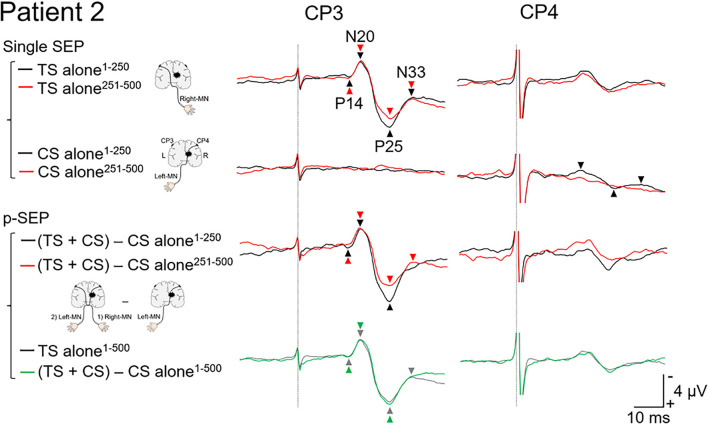
Paired median nerve somatosensory evoked potential in patient 2. *Left panels*: to investigate the effect of conditioning stimulus (CS) on the SEP induced by the test stimulus (TS), the single median nerve SEPs (single SEP) (left-MN: CS alone), and the paired median nerve SEPs (p-SEP) were recorded at the CP3 and CP4 electrodes (Ragert et al., 2011). In the p-SEP recording, the left-MN stimulus (CS) was performed 1 ms after the right-MN stimulus (TS). *Right panels*: all SEP waveforms at CP3 and CP4 from patient 2. We compared the SEP averaged over the first 250 epochs with the SEP averaged over the second 250 epochs (total number of epochs recorded: 500). Black line: the SEP averaged over the first 250 epochs. Red line: the SEP averaged over the second 250 epochs. Gray line: the SEP averaged over all epochs (500 epochs) (TS alone condition). Green line: the SEP averaged over all epochs (500 epochs) [(TS + CS) − CS alone condition]. Dotted line: the time duration of stimulation. MN, median nerve.

To investigate the interactions between contralateral and ipsilateral activations, single SEP (s-SEP), and p-SEP were recorded using a previously described protocol with minor modifications (Ragert et al., 2011; Brodie et al., 2014; Ishii et al., 2021). In the s-SEP paradigm, s-SEPs were obtained by stimulation of the right and left median nerves at the wrist (Figures 2, 3). In the p-SEP paradigm, peripheral stimulation to the median nerve of the affected side (conditioning stimulus: CS) was performed 1 ms after the stimulation to the non-affected side (test stimulus: TS). A BrainAmp DC (Brain Products, Gilching, Germany) and TMS-compatible EEG electrode caps were used for SEP recordings. The ground electrode was placed on the skin over the right biceps brachii. The impedance of each electrode was set below 5 kΩ. EEG signals were recorded at a sampling frequency of 5 kHz with 1 kHz low-pass analog filter.

A Neuropack X1 (Nihon Kohden, Tokyo, Japan) was used to deliver electrical stimuli of 0.2 ms duration at a rate of 3 Hz (Ishibashi et al., 2020). The stimulus intensity was set at three times the perceptual threshold of the non-affected side (patient 1: 10.5 mA for bilateral median nerve; patient 2: 11.4 mA for bilateral median nerve). During stimulation for s-SEP, TS (non-affected side), and CS (affected side) were applied to the median nerve. A total of 1,500 stimulus-related epochs were recorded with 500 epochs for each condition [(TS + CS), TS only, and CS only]. We asked the subjects to count the electrical stimuli during the SEP recordings to counteract the attention effect.

### Data Analysis

Epochs were digitally filtered using a bandpass Butterworth filter (1–200 Hz) (Ragert et al., 2011; Brodie et al., 2014; Ishii et al., 2021). We calculated and compared the average of the SEP from 1 to 250 epochs with those from 251 to 500 epochs (Figures 2, 3). Each SEP component was calculated using the montages described below using MATLAB R2019a (MathWorks, Natick, MA, United States) (Mauguiere et al., 1999; Cruccu et al., 2008). The cortical components of short latency SEP (P14-peak–N20-peak amplitude, N20-peak–P25-peak amplitude, and P25-peak–N33-peak amplitude) from CP3 or CP4 (Fz reference) were calculated.

To evaluate the effect of the CS (stimulation to the affected side) on TS (stimulation to the non-affected side), the p-SEPs in the contralateral and ipsilateral pathways (data at CP3 and CP4, respectively) were calculated using the following equation (Ragert et al., 2011; Brodie et al., 2014; Ishii et al., 2021):


p-SEP=(SEPofTS+CS)-(SEPofCSonly)

## Results

### Motor Paralysis and Sensory Impairment at the Day of SEP Recording

#### Patient 1

Brunnstrom stages of the paretic limbs were 3 for arm, 4 for hand, and 4 for leg, respectively. Hypoesthesia was severe in deep (sense of passive movement and position sense of thumb) and superficial sensations (Figure 1B). During the evaluation of deep sensation, the patient remarked, “I can vaguely tell that it is moving, but I cannot identify the direction or the finger that is moving.” Moreover, numbness and pain in the upper limb on the affected side increased since admission to the convalescent rehabilitation hospital.

#### Patient 2

Brunnstrom stages of the paretic limbs were 5 for arm, 5 for hand, and 4 for leg. Hypoesthesia was mild in deep and superficial sensations (Figure 1B). At the time of the SEP recording, the patient said, “I felt the sensation more strongly with (the single than) the paired stimulation.” Moreover, he complained of numbness in the ball of the left thumb and no pain in the affected upper limb.

### Evaluation of Somatosensory Pathway After Stroke

#### Patient 1

In the TS alone condition (non-affected side) at CP4 (Figure 2), the amplitude of N20/P25 averaged over the second half (251–500 epochs; 9.5 μV) was smaller than that over the first half (1–250 epochs; 11.2 μV), despite the other components showing less difference between the first (P14/N20, 3.4 μV and P25/N33, 6.8 μV) and second halves (P14/N20, 3.7 μV and P25/N33, 7.0 μV). In the CS alone condition (affected side) at CP3, no cortical components were recorded. After subtracting the CS alone condition from the TS + CS condition for each half, a small difference was observed between the first (P14/N20, 3.7 μV; N20/P25, 11.7 μV; and P25/N33, 7.9 μV) and second halves (P14/N20, 3.1 μV; N20/P25, 11.7 μV; and P25/N33, 8.0 μV). However, in terms of the average over 1–500 epochs, the amplitude of the N20/P25 (11.6 μV) and P25/N33 (7.8 μV) for “(TS + CS) − CS alone” was larger than that for TS alone (N20/P25, 10.4 μV and P25/N33, 6.9 μV). On the other hand, there was less difference between the TS alone (3.6 μV) and p-SEP (3.3 μV).

#### Patient 2

In the TS alone condition at CP3 (Figure 3), the amplitudes of the second half (N20/P25, 7.1 μV and P25/N33, 2.4 μV) were extremely smaller than those of the first half (N20/P25, 8.0 μV and P25/N33, 3.6 μV). On the other hand, in the P14/N20 component, a small difference was observed between the first (1.9 μV) and second half (2.1 μV). In the “(TS + CS) − CS” conditions at CP3, the amplitudes of the second half (N20/P25, 6.8 μV) were extremely smaller than those of the first half (N20/P25, 9.1 μV). In the P14/N20 component, few differences between the first (2.5 μV) and second half (1.8 μV) were observed. N33 component of the second half could not be identified due to uncertainty. These results of the “(TS + CS) − CS” condition were different from that of patient 1. Consequently, in the average over 1–500 epochs, there were few differences between the TS alone (P14/N20, 2.0 μV and N20/P25, 7.6 μV) and “(TS + CS) − CS” (P14/N20, 2.2 μV and N20/P25, 7.9 μV) conditions. In the CS alone condition at CP4, the cortical components recorded in the first half (P14/N20, 0.7 μV; N20/P25, 2.3 μV; and P25/N33, 0.6 μV) disappeared in the second half.

## Discussion

In this case report, we investigated the interaction between contralateral and ipsilateral activations in the somatosensory pathways in two patients with thalamic hemorrhage. When the left and right somatosensory pathways are directly connected, the ipsilateral SEPs would be recorded (Noachtar et al., 1997). Additionally, the amplitude of these ipsilateral SEPs evoked by the second median nerve stimulus (affected side) would be attenuated or eliminated (during the refractory period) by the first median nerve stimulus (non-affected side) under short ISI conditions (e.g., 1–5 ms). No cortical components had been previously identified when the ISI of the paired stimulation to the same median nerve was short (Hoshiyama and Kakigi, 2002). In the present case report, no ipsilateral SEPs were recorded from the two patients (Figures 2, 3).

In patient 1, the amplitude of N20/P25 of the p-SEP with bilateral median nerve stimulation was larger than that of the s-SEP with unilateral median nerve stimulation. On the other hand, in patient 2, the amplitudes of N20/P25 of the p-SEP and s-SEP were of the same degree. These results suggest that left and right somatosensory pathway may have formed a connection in patient 1. Moreover, to investigate this phenomenon in more detail, we calculated and compared the average of the SEP from 1 to 250 epochs with the average of the SEP from 251 to 500 epochs (Figures 2, 3). As a results, in patients 1 and 2, the amplitude of N20/P25 with continuous stimulation to the non-affected side was smaller in the second half than in the first half. In addition, in patent 2, after subtracting the CS alone condition (left median nerve stimulation) from the TS + CS condition (p-SEP), the amplitude of N20/P25 was smaller in the second half than in the first half. On the other hand, in patient 1, the amplitude of the p-SEP “(TS + CS) − CS” in the first and second half were same level. We have previously reported that continuous electrical stimulation of the median nerve decreases the amplitude of N20/P25 of the SEPs (Ishibashi et al., 2021a). This may be caused by habituation to continuous electrical stimulation, resulting in an attenuated central nervous system response to electrical stimulation. In current case report and our previous study, the patient 1 had left–right interaction after thalamic hemorrhage, and somatosensory input from the affected side interfered with the habituation of the contralateral somatosensory system, and conversely increases the response.

In mild cases of thalamus injury, the amplitude of N20/P25 of the SEP induced by the stimulation of the affected side was attenuated when the non-affected side was stimulated by vibration in the early stage (Staines et al., 2002). This indicates that patients with thalamocortical stroke have reduced ability to gate competing sensory information from the non-affected side when sensory stimulation is applied to both affected and non-affected sides. In addition, the decrease in SEPs is normalized with recovery of symptoms. Patient 1 in our case report was a severe case, and it is possible that the abnormalities in the gating mechanisms of left and right somatosensory pathway persist.

The difference between patients 1 and 2 was that patient 2 had better recovery of sensory deficits than patient 1. As for other symptoms, patient 1 had pain in the affected upper limb, but patient 2 did not complain of pain. Chronic pain caused by the thalamic nucleus after stroke is the maladaptive plasticity of the central nervous system that constitutes a pain-related network (Willoch et al., 2004; Seghier et al., 2005; Kumar et al., 2009; Casey et al., 2012; Ohn et al., 2012; Krause et al., 2016; Nagasaka et al., 2021). Based on this information and the SEP results, it is possible that interactions between the left and right somatosensory pathways observed in a patient with thalamic hemorrhage are related to pain rather than recovery. However, the details are still unclear because other factors have not been thoroughly investigated.

This case report has several limitations. First, we were unable to identify the factors causing the interaction between the left and right somatosensory pathway in patient 1 because patients 1 and 2 had different degrees of recovery and symptoms (e.g., presence of pain) as well as different hemispheres of injury. A large body of evidence from studies on healthy individuals and patients with brain injury shows that structural and functional asymmetry exists between the left and right hemispheres of the human brain (Kinsbourne, 1977; Corbetta et al., 1993; Thiebaut de Schotten et al., 2011; Duecker and Sack, 2015; Ishii et al., 2019; Ishibashi et al., 2021b). In addition, 5 months had passed since the onset of symptoms in patient 2, and about 3 months had passed since the onset of symptoms in patient 1. Future studies involving a large number of patients are necessary to establish the presence or absence of the left–right interaction of somatosensory pathways after stroke. Second, we have not been able to identify the site where the left and right somatosensory pathways were connected. The current case report indicates that damage to the thalamus may cause neuroplasticity in the left–right interaction (e.g., left and right S1). The somatosensory input from the affected side may interfere with the habituation of the contralateral somatosensory system and conversely increase the response. Third, the blindness of evaluators of SEPs was incomplete. Finally, only patients with thalamic hemorrhage were included in this study. Further validation is needed, which can be achieved by applying the results of this study to patients with central nervous system injury.

## Data Availability Statement

The original contributions presented in the study are included in the article/supplementary material, further inquiries can be directed to the corresponding author.

## Ethics Statement

This study was reviewed and approved by the Ibaraki Prefectural University of Health Sciences Review Board (approval nos. 893 and e278). The patients/participants provided their written informed consent to participate in this study. Written informed consent was obtained from the individual(s) for the publication of any potentially identifiable images or data included in this article.

## Author Contributions

DI, KI, KT, HY, AY, and YKo: conceptualization. DI, KI, KT, and YKa: methodology and investigation. DI, KT, and SY: formal analysis. DI: writing – original draft. KI, KT, HY, SY, YKa, AY, and YKo: writing – review and editing. All authors contributed to the article and approved the submitted version.

## Conflict of Interest

The authors declare that the research was conducted in the absence of any commercial or financial relationships that could be construed as a potential conflict of interest.

## Publisher’s Note

All claims expressed in this article are solely those of the authors and do not necessarily represent those of their affiliated organizations, or those of the publisher, the editors and the reviewers. Any product that may be evaluated in this article, or claim that may be made by its manufacturer, is not guaranteed or endorsed by the publisher.
